# Comparison of the physicochemical properties of palm-based shortenings of various melting temperatures and animal fats

**DOI:** 10.1016/j.fochx.2025.102583

**Published:** 2025-05-26

**Authors:** Mohd Razali Faridah, Amelia Najwa Ahmad Hairi, Masni Mat Yusoff, Ashari Rozzamri, Wan Zunairah Wan Ibadullah, Mohammad Rashedi Ismail-Fitry

**Affiliations:** a Department of Food Technology, Faculty of Food Science and Technology, Universiti Putra Malaysia, 43400 UPM Serdang, Selangor, Malaysia; b SD Guthrie Technology Centre Sdn. Bhd., 1st Floor, Block B, UPM-MTDC, Technology Centre III Malaysia, Lebuh Silikon, Putra Square, 43400 Serdang, Selangor, Malaysia; c Halal Products Research Institute, Universiti Putra Malaysia, 43400 UPM Serdang, Selangor, Malaysia; d Department of Food Science, Faculty of Food Science and Technology, Universiti Putra Malaysia, 43400 UPM Serdang, Selangor, Malaysia

**Keywords:** Lipid profiles, Thermal behaviour, Comparative analysis, Fat substitutes, Chemometrics

## Abstract

This study compares the physicochemical properties of palm-based shortenings (PS) with varying melting temperatures (33–60 °C) to animal fats from chicken, beef, and mutton. Key parameters assessed include peroxide value, solid fat content, thermal behaviour, fatty acid composition, and crystal structure. PS exhibits lower peroxide values (0.45–8.33 meq/kg) than animal fats (5.02–12.21 meq/kg), indicating greater oxidative stability. PS with higher melting points (55–60 °C) demonstrates enhanced solid fat content (up to 98 % at 10 °C) and thermal stability, while lower-melting PS (33–42 °C) displays β’ crystal dominance, closely resembling the texture of animal fats. Principal component analysis reveals that chicken fat clusters with PS of 33–49 °C, suggesting that these PS can directly substitute animal fats in food applications. Palm-based shortenings with melting points ranging from 33 to 49 °C exhibit functional and structural characteristics that position them as effective and sustainable alternatives to animal fats (particularly chicken fat).

## Highlights

• Palm-based shortenings demonstrated superior oxidative stability to animal fats.
• Palm-based shortening with 55–60 °C melting points provided high solid fat content and thermal stability.
• Principal component analysis revealed that chicken fat clustered with low-melting palm shortenings.
• Lower-melting PS mimicked animal fat crystallinity and texture in foods.
• Palm-based shortenings offered sustainable alternatives to animal fats.

## 1 Introduction

Shortening consists of edible fats formulated from either vegetable or animal lipids. These lipids must undergo several processing steps to achieve a specific composition tailored to their intended application. Consequently, the melting temperatures of the shortening can vary based on their compositions. Palm-based fat is an ideal raw material for producing shortening due to its texture (solid at room temperature), stability (featuring a favourable saturated fatty acid to unsaturated fatty acid ratio, which enhances stability and resistance to oxidation), and neutral flavour profile (to prevent flavour overpowering in final products) (Sulaiman et al., 2022). Furthermore, palm-based fat is viewed as an economically advantageous choice for producing shortenings because of its cost-effectiveness, versatility, and substantial contribution to export revenues. At the same time, sustainability is increasingly ensured through Roundtable on Sustainable Palm Oil (RSPO) certification, which promotes environmental conservation, social benefits for smallholders, and climate change mitigation across the global palm oil supply chain (Roundtable on Sustainable Palm Oil, 2024).

Animal fats, derived from animal adipose tissues, have been used in food preparation since the 18th century. Despite their historical significance, animal-based fats remain relevant in food applications due to their texture and flavour-enhancing properties, which provide energy and improve the digestibility of fat-soluble vitamins (Woodgate & Van Der Veen, 2004). However, the use of animal fats in food manufacturing raises several concerns. Notably, the supply of animal fats can be inconsistent due to fluctuations in the livestock industry. Furthermore, issues regarding sustainability, environmental impacts, and ethical implications related to animal fat production are increasingly pertinent (Bryant, 2022). Additionally, certain religious dietary restrictions limit the consumption of specific animal-based products, such as beef fat for Hindus and pork derivatives for Muslims, further complicating their use in diverse food systems.

The melting point of fats significantly influences their physicochemical and functional properties, including texture, crystallinity, and thermal behaviour, which affect their performance in food products. Animal fats, such as chicken, beef, and mutton, have distinct melting profiles that determine their suitability for various applications. Palm-based shortenings can be tailored through fractionation and blending to achieve a broad range of melting points, allowing them to mimic or enhance these properties. For instance, meat emulsions formulated with palm shortenings of higher melting ranges (around 55–60 °C) exhibit physicochemical and textural characteristics similar to those made with chicken fat, making them suitable for comparable uses (Faridah et al., 2023). Additionally, cookies made with lower melting points (45 °C) of palm shortenings have increased solid fat content and thermal stability, which improve product hardness and moisture retention, comparable to conventional shortenings used in baking (Perez-Santana et al., 2022). This literature evidences the adaptability of palm-based shortenings to mimic or enhance animal fat functionality through targeted melting point adjustments. Therefore, this study aims to determine the optimal melting temperatures of the palm-based shortenings that most effectively replicate the functional properties of either chicken, beef, and mutton fats for sustainable food applications through a comprehensive comparison of their physicochemical characteristics.

## 2 Materials and methods

### 2.1 Sample preparation

Five palm shortenings with different melting temperatures (33–36 °C, 38–42 °C, 44–46 °C, 45–49 °C, and 55–60 °C) were provided by SD Guthrie Technology Centre Sdn. Bhd., Selangor, Malaysia. Animal fats (chicken, beef, and mutton) were purchased from a local wet market in Pasar Borong Sri Kembangan, Selangor, Malaysia. All animal fats were ground separately using a mincer machine (Model 4822, Hobart, Offenburg, Germany). The chicken fat was extracted from the ground chicken skin, while the beef and mutton fats were obtained from the adipose tissue of various parts of the respective animals. The animal fats were freeze-dried at a temperature of −50 °C for five days before the extraction process. The extraction was performed using supercritical fluid extraction (SCFE) Lab Scale Plant (Model SFE-1000, Deven Supercriticals Pvt. Ltd., Mumbai, India) at a pressure of 30 MPa and a temperature of 50 °C, as described by Ahmadkelayeh et al. (2022). All samples were stored in airtight containers at 4 °C before physicochemical analyses.

### 2.2 Reagents

All reagents for this work were of analytical grade. All chemicals used, i.e. acetic acid (99.8 %), potassium iodide (99.5 %), sodium thiosulphate (99.5 %), isooctane (99.8 %), glacial acetic acid (99.8 %), anisidine reagent (99 %), isopropanol (99.5 %), sodium hydroxide (98 %), cyclohexane (99.5 %), Wijs solution, and sodium thiosulphate (99.5 %), were supplied by Merck (Darmstadt, Germany).

### 2.3 Peroxide value (PV)

The peroxide value was determined according to the Malaysian Palm Oil Board (MPOB) Test Method p2.3 (Malaysian Palm Oil Board, 2004), which is technically equivalent to the AOCS Official Method Cd 8b-90 for determining the peroxide value in refined and unrefined edible oils. Firstly, 0.3 g of the sample was placed into a conical flask with a stopper. Fifty millilitres of acetic acid were added, and the flask was swirled until the sample dissolved completely. Approximately 0.5 mL of saturated potassium iodide was added. The solution was allowed to stand for 1 min, thoroughly shaken thrice during this period. Next, 30 mL of distilled water was added to the solution. The solution was then titrated with sodium thiosulphate solution with constant and vigorous agitation until the yellow iodine colour had almost disappeared. The titration was continued by adding 0.5 mL of starch solution while maintaining constant agitation to liberate all of the iodine from the solvent layer. The sodium thiosulphate solution was added drop by drop until the blue colour disappeared. The blank was carried out in the same manner without the fat sample. The peroxide value was calculated according to the underlying formula:

(1) $$\mathrm{Peroxide value}\;\left(\mathrm{meq}/\mathrm{kg}\right)=\frac{1000\times \left(S-B\right)\times C}{\mathrm{Weight of sample}\;\left(g\right)}$$

where,

S = the volume of sodium thiosulphate used for the determination, in mL;
B = the volume of sodium thiosulphate used for the blank determination, in mL; and.
C = the concentration of the sodium thiosulphate, in moles per litre.

### 2.4 Anisidine value (AV)

The anisidine value was determined using the Malaysian Palm Oil Board (MPOB) Test Method p2.4 (Malaysian Palm Oil Board, 2004), technically equivalent to ISO 6885:2016, the standard method for determining anisidine value in animal and vegetable fats and oils. Firstly, 1 mg of the sample was weighed into a 25-mL volumetric flask, and isooctane was added to achieve a final volume of 25 mL. Next, 5 mL of the diluted solution was transferred into a test tube. 1 mL of glacial acetic acid was added to the tube, which was then closed with a stopper and shaken well. The test tube was incubated in the dark at 23 ± 3 °C for 10 min. The solution was then transferred into a dry spectrometer cell, and absorbance was measured (A0) at 350 nm. In another test tube, 5 mL of the diluted sample was added, followed by the addition of 1 mL of anisidine reagent. This test tube was closed with a stopper and shaken well. It was also incubated in the dark at 23 ± 3 °C for 10 min. The solution was then transferred into a dry spectrometer cell, and absorbance was measured (A1) at 350 nm (GENESYS 10S UV–Vis Spectrophotometer, Thermo Fisher, Massachusetts, USA). To prepare the blank, 5 mL of isooctane was transferred into a test tube, and 1 mL of the anisidine reagent was added. The test tube was closed with a stopper and shaken well, then incubated in the dark at 23 ± 3 °C for 10 min. The solution was transferred into a dry spectrometer cell, and absorbance was measured (A2) at 350 nm. The anisidine value was calculated using the following expressions:

(2) $$\mathrm{AnV}=\frac{100\times Q\times V\times 1.2\left[\left(A1-A2-A0\right)\right]}{m}$$

where

Q = the sample content of the measured solution, in grams per millilitre (Q = 0.1 g/mL);
V = the volume in which the test sample is dissolved, in millilitres (V = 25 mL);
m = the mass of the test portion, in grams;
A0 = the absorbance of the unreacted test solution;
A1 = the absorbance of the reacted solution;
A2 = the absorbance of the blank; and
1.2 = the correction factor for the dilution of the 5 mL of the test solution with 1 mL of the reagent.

### 2.5 Free fatty acid (FFA)

The samples were tested for acidity using the Malaysian Palm Oil Board (MPOB) Test Method p2.5 (Malaysian Palm Oil Board, 2004), which is technically equivalent to the AOCS Official Method Ca 5a-40 for commercial fats and oils. First, 5 g of the sample was weighed into a conical flask. Then, 50 mL of pre-neutralised isopropanol was added to the sample. The flask was subsequently placed on a hot plate and heated until it reached a temperature of 40 °C. The mixture was titrated with sodium hydroxide solution (0.1 N) while the flask was gently shaken until the first permanent colour appeared for at least 30 s. The FFA% was determined using the following formula.

(3) $$\mathrm{FFA}\;\left(\%\right)=\frac{2.6\times M\times V}{m}$$

where

M = the molarity of the standard sodium hydroxide solution;
B = the volume of the standard sodium hydroxide solution used, in mL; and.
m = the mass of the sample in grams.

### 2.6 Rancimat induction period test

The induction times of the animal fats and palm-based shortening samples were measured by the Rancimat method following the AOCS method Cd 12b-92 (AOCS, 1997). The measurement was completed using a Metrohm 743 Rancimat instrument (Metrohm AG, Herisau, Switzerland), with fixed operational parameters for temperature, sample weight, and airflow rate set at 100 °C, 3.9 g, and 25 L-h, respectively.

### 2.7 Fatty acid composition

The fatty acid composition was determined using the gas chromatography method outlined in the Malaysian Palm Oil Board (MPOB) Test Method p3.5 (Malaysian Palm Oil Board, 2004). The fatty acids from palm-based shortening samples were initially converted to fatty acid methyl esters (FAME) following the procedure described in MPOB Test Method p3.4-Part 1 (Malaysian Palm Oil Board, 2004), while the fatty acids from animal fats were converted according to AOCS Official Method Ce 2–66 (AOCS, 2013). Each FAME sample was then analysed with a gas chromatograph system equipped with a flame ionisation detector (Shimadzu, Tokyo, Japan) and an open tubular fused silica capillary column (0.25 mm i.d x 60 m) to obtain the fatty acid profiles. The injector and detector temperatures were maintained at 240 °C, while the oven temperature was initially set at 120 °C, then increased to 185 °C at a rate of 3 °C/min. Helium was used as the carrier gas at a flow rate of 0.8 mL/min, and the injection volume was 1 μL.

### 2.8 Iodine value

Iodine value was determined using the method reported in Malaysian Palm Oil Board (MPOB) Test Method p3.2 (Malaysian Palm Oil Board, 2004), equivalent to the ISO 3961:2018 standard for determining iodine value in animal and vegetable fats and oils. All samples were initially melted at temperatures specific to their respective melting temperature ranges. Four mL of the melted sample was transferred into a 500 mL conical flask. Fifteen mL of cyclohexane and glacial acetic acid were added, ensuring the sample was completely dissolved. Then, twenty-five mL of Wijs solution was dispensed into the conical flask, which was then closed with a stopper and swirled thoroughly to ensure an intimate mixture. The mixture was kept in the dark for 30 min at a temperature of 25 °C. Twenty mL of potassium iodide solution was added, followed by 100 mL of distilled water, and the mixture was well mixed. The resulting mixture was titrated against 0.1 mol/L sodium thiosulphate solution, using starch as an indicator, with vigorous shaking to extract iodine from the chloroform layer. The blank was conducted similarly in the absence of the sample. The iodine value was calculated according to the following equation:

(4) $$\mathrm{Iodine value}=\frac{\left(B-S\right)\times M\times 12.69}{\mathrm{Weight of sample}\;\left(g\right)}$$

where

B = the volume of sodium thiosulphate used for the titration of the blank, in mL;
S = the volume of sodium thiosulphate used for the titration of the sample, in mL; and.
M = the molarity of sodium thiosulphate.

### 2.9 Solid fat content

SFC was measured according to the Malaysian Palm Oil Board (MPOB) Test Method p4.8 (Malaysian Palm Oil Board, 2004), which is technically equivalent to the AOCS Official Method Cd 16b-93 using time-domain nuclear magnetic resonance (TD-NMR) spectrometry (Bruker, Zhubei, Taiwan). The SFC of all fat samples was measured at each separation temperature. The sample was melted at 70 °C, mixed thoroughly, and subsequently transferred into a sample tube to a height of 3 cm. The sample tube was placed in a water bath and kept at 70 °C for 30 min. Next, the temperature of the water bath was adjusted to 0 °C, and the sample tube was maintained for 90 min before measurement. After measuring the percentage of SFC at 0 °C, the water bath temperature was set to the next measuring temperature. The sample's melting, chilling, and holding processes were repeated in the same manner as described and conducted in a pre-equilibrated thermostatic water bath. The SFC measuring temperatures were set to 0, 10, 20, 25, 30, 35, 40, 45, 50, 55, and 60 °C.

### 2.10 Lovibond colour profile

The Lovibond colour of the sample was determined using the Malaysian Palm Oil Board (MPOB) Test Method p4.1 (Malaysian Palm Oil Board, 2004), which is equivalent to ISO 15305:1998, utilising a Lovibond Universal Tintometer Model F (Tintometer GmbH, Dortmund, Germany). All samples were first melted at temperatures according to their respective melting temperature ranges. The melted sample was poured into a glass cell up to three-quarters full and placed within the lighting cabinet. The lid of the lighting cabinet was closed, and the colour of the sample was immediately determined by using the colour racks equipped with the standard colour slides until an accurate colour match was obtained.

### 2.11 Slip melting point (SMP)

SMP was measured according to the Malaysian Palm Oil Board (MPOB) Test Method p4.2 (Malaysian Palm Oil Board, 2004), which is equivalent to the ISO 6321:2021 method. All samples were first melted and filtered through filter paper. The filtered samples were left in the oven for 10 min to remove air bubbles. Clean capillary tubes were dipped into the melted sample to obtain an approximately 10 mm high fat column. The fat columns were immediately chilled by holding and rolling the ends of the tubes against ice until solidification. Care was taken to avoid contact between the open ends of the tubes and the ice. The tubes were wiped with tissue to remove excess fat from the exterior, then placed in a test tube held in a beaker of water equilibrated at 10 ± 1 °C in a thermostatic water bath (BIOBASE, Jinan, China). The beaker remained in the bath for 16 h at 10 ± 1 °C. After conditioning, the capillary tubes were removed and attached to a thermometer with a rubber band, ensuring the tube ends were level with the bottom of the thermometer's mercury bulb. The thermometer and tubes were suspended in a beaker containing 400 mL of boiled distilled water, with the lower end of the thermometer immersed to a depth of approximately 30 mm. The water was agitated with a magnetic stirrer, and the temperature was increased at 1 °C per min until the fat column in each tube rose. The SMP was recorded as the temperature of the water when each fat column rose.

### 2.12 Differential scanning calorimetry (DSC)

The thermal properties of all samples were measured using a Mettler Toledo differential scanning calorimeter (DSC 823 Model, Columbus, OH, USA), and the data were analysed using thermal analysis software (STARe software, Version 9.0×, Schwerzenbach, Switzerland). An empty, hermetically sealed DSC aluminium pan was employed as a reference. Approximately 5–10 mg of the sample was placed in the pan, hermetically sealed, and kept at 25 °C for 1 min. Next, the temperature was rapidly raised to 70 °C at 10 °C/min and held for 10 min to ensure complete melting and obtain the melting profiles. Then, the sample was cooled to −50 °C at a rate of 10 °C/min to obtain the cooling profiles. The procedures refer to Zhang et al. (2021), with some modifications.

### 2.13 The X-ray diffraction (XRD)

The crystallinity of the samples was observed using an X-ray diffractometer (SHIMADZU XRD-6000, Japan). The samples were scanned at a diffraction angle of 2θ ranging from 5° to 60°, with a scanning rate of 2 °C/min. The polymorphic forms were analysed using X'pert High Score Plus software (Asyrul-Izhar et al., 2023).

### 2.14 Statistical analysis

#### 2.14.1 Analysis of variance (ANOVA)

The data analysis of variance (ANOVA) was performed using Minitab Statistical Software Version 19 (Minitab Inc., State College, PA, USA). All data obtained from the experimental measurements (except for DSC and XRD) were subjected to one-way ANOVA to determine the significant differences among the samples defined at *p* < 0.05. All measurements (except for DSC and XRD) were conducted in triplicate and reported as the Mean ± SD of independent trials. Significant differences (*p* < 0.05) between means were subsequently determined by Tukey's test.

#### 2.14.2 Principal Component Analysis (PCA)

The multivariate method of principal component analysis (PCA) was performed using the data of physicochemical properties as a matrix variable to deduce similarities among the palm-shortening and animal fat samples. All prerequisite analyses before the PCA, which included (1) removing outliers using a box and whisker plot, (2) confirming the dataset's adequacy using the Kalser-Meyer-Olkin test, and (3) transforming the data into a normal distribution through (n-1) standardization, were conducted according to Ismail et al. (2021). The prerequisite analysis and PCA were executed using XLSTAT-Pro (2019) statistical software (Addinsoft, Paris, France).

## 3 Results and discussion

### 3.1 Oxidative stability (PV, AV, FFA, and Rancimat induction period)

Peroxide value (PV) and anisidine value (AV) are the most widely used tests for measuring the oxidative status of oil and fat samples. These tests are important for assessing the quality and rancidity of fat samples. The PV determines the primary oxidation of hydroxyl groups in unsaturated fatty acids by measuring the levels of peroxides and hydroperoxides in the samples, while AV evaluates secondary oxidation by measuring the aldehyde and ketonic breakdown products of peroxides (Gilbraith et al., 2021; Talbot, 2016). The lower the values of these parameters, the higher the quality of the fat samples. Among all the fat samples tested, sample 55–60 revealed better oxidative strength, exhibiting significantly lower PV and AV values compared to the other fat samples. This can be inferred from the higher proportion of saturated fatty acids (SAFA) in sample 55–60 (see Table 2), which are more stable to oxidation than samples with a higher proportion of unsaturated fatty acids. This aligns with findings by Marcus (2013) and Vieira et al. (2015), who note that SAFA-rich fats resist oxidation more effectively, a critical advantage for applications like frying or baked goods with extended shelf-life requirements.

The determination of free fatty acid (FFA) content is one of the important analyses for assessing the quality of fats and oils. The FFA value measures the hydrolytic rancidity of the raw materials. Therefore, a lower FFA value is desirable for food products, as it indicates higher quality and a longer shelf life (Muzolf-Panek & Kaczmarek, 2021). According to Table 1, FFA analysis further supports PS viability: animal fats exhibited significantly higher FFA values (up to 7.31 % in mutton fat) compared to PS (≤0.41 %), which remained compliant with the Malaysia Food Regulations 1985. In addition, the Rancimat induction period test is one of the methods used to study fat oxidative stability. Theoretically, a longer induction period indicates that the fat is more stable and less likely to go rancid, while a shorter period notes the opposite (Talbot, 2016). As shown in Table 1, animal fats had a significantly lower induction period (0.07–0.41 h) compared to palm-based shortenings (10.92–13.85 h). The results indicate that animal fats are more prone to oxidising and rancid than palm-based shortenings.

**Table 1:** Oxidative stability attributes of palm-based shortenings (PS) of various melting temperatures (MT) and animal-based fats.

Sample	PV (meq/kg)	AV	FFA (%)	Rancimat induction period (h)
33–36	3.50 ± 0.22^g^	1.44 ± 0.09^abc^	0.29 ± 0.00^de^	13.85 ± 0.13^b^
38–42	5.64 ± 0.07^e^	2.97 ± 0.15^a^	0.05 ± 0.00^f^	10.92 ± 0.16^d^
44–46	8.33 ± 0.38^c^	1.17 ± 0.10^bc^	0.04 ± 0.00^f^	13.35 ± 0.16^c^
45–49	7.61 ± 0.25^d^	1.45 ± 0.29^abc^	0.13 ± 0.00^ef^	13.36 ± 0.10^c^
55–60	0.45 ± 0.03^h^	0.27 ± 0.06^c^	0.41 ± 0.00^d^	114.64 ± 0.00^a^
CF	11.62 ± 0.15^b^	0.77 ± 0.46^bc^	1.87 ± 0.21^c^	0.07 ± 0.01^f^
BF	12.21 ± 0.15^a^	1.51 ± 0.43^abc^	7.30 ± 0.01^a^	0.02 ± 0.01^f^
MF	5.02 ± 0.19^f^	2.19 ± 1.51^ab^	2.60 ± 0.03^b^	0.41. ± 0.01^e^

The values above are expressed in mean ± SD (*n* = 3). Different superscript letters (^a–h^) within the same column indicate significant differences (*p* < 0.05, Tukey's test). PV = Peroxide value; AV = Anisidine value; FFA = Free fatty acid; 33–36 = PS at MT of 33–36 °C; 38–42 = PS at MT of 38–42 °C; 44–46 = PS at MT of 44–46 °C; 45–49 = PS at MT of 45–49 °C; 55–60 = PS at MT of 55–60 °C; CF = Chicken fat; BF = Beef fat; MF = Mutton fat.

The analyses conducted to evaluate the oxidative stability of the fat samples indicate that palm-based shortenings exhibit superior oxidative resistance compared to animal fats. While specific industry standards for ideal peroxide value, acid value, free fatty acid content, and Rancimat induction period are not established, as these parameters vary depending on the application and type of fat, it is evident that palm-based shortenings with diverse melting temperatures offer enhanced oxidative stability and quality relative to animal fats. This makes them a promising option for sustainable food production. The stability of these shortenings is critical for preserving the nutritional value and safety of high-fat food products, potentially mitigating the risk of metabolic disorders associated with oxidised fats.

### 3.2 Fatty acid composition and iodine value

Table 2 displays the comprehensive fatty acid composition of all palm-based shortening and animal fat samples. The results for the animal fats were all within the ranges reported in previous studies (Nizar et al., 2013; Nurjuliana et al., 2011). The results showed that all fat samples had higher proportions of saturated fatty acids (SAFAs) than unsaturated fatty acids (USFAs), except for CF. The saturated fatty acids in all fat samples were predominantly composed of palmitic acid (C16:0) and stearic acid (C18:0), while appreciable amounts of myristic acid (C14:0) were also recorded. Samples 55–60 exhibited a remarkable quantity of SAFAs, showing a staggering 99.66 % (58.29 ± 0.04 % palmitic acid and 39.37 ± 0.08 % stearic acid) of the overall amount of fatty acids. On the other hand, the USFAs of all samples consisted mainly of oleic acid (C18:1) and linoleic acid (C18:2), with small proportions of α-linolenic acid (C18:3) and eicosenoic acid (C20:1) also observed. CF recorded the highest amount of USFAs, as much as 68.96 %, dominated by oleic acid (39.92 ± 0.20 %) and linoleic acid (20.79 ± 0.08 %).

**Table 2:** Fatty acids composition and iodine values of palm-based shortenings (PS) of various melting temperatures (MT) and animal-based fats.

Fatty acid composition (%)	33–36	38–42	44–46	45–49	55–60	CF	BF	MF
C10	n.d	n.d	0.01 ± 0.01^b^	n.d	n.d	n.d	n.d	0.13 ± 0.11^a^
C12	0.20 ± 0.00^bcd^	0.13 ± 0.11^d^	0.20 ± 0.00^bcd^	0.19 ± 0.00^cd^	0.30 ± 0.02^b^	0.28 ± 0.01^bc^	1.51 ± 0.01^a^	0.19 ± 0.00^bcd^
C14	1.06 ± 0.01^d^	1.05 ± 0.02^d^	1.16 ± 0.01^c^	1.08 ± 0.01^d^	1.20 ± 0.01^c^	1.02 ± 0.02^d^	7.92 ± 0.06^a^	3.36 ± 0.01^b^
C14:1	n.d	n.d	n.d	n.d	n.d	n.d	0.91 ± 0.01^a^	0.48 ± 0.00^b^
C15	n.d	n.d	n.d	n.d	n.d	n.d	0.93 ± 0.01^a^	0.88 ± 0.01^b^
C16	44.18 ± 0.23^e^	44.29 ± 0.27^d^	48.52 ± 0.13^c^	53.27 ± 0.11^b^	58.29 ± 0.04^a^	24.76 ± 0.04^g^	30.29 ± 0.05^f^	23.56 ± 0.11^h^
C16:1	0.19 ± 0.09^d^	0.19 ± 0.03^d^	0.16 ± 0.05^d^	0.10 ± 0.00^d^	0.15 ± 0.01^d^	6.42 ± 0.10^a^	3.50 ± 0.02^b^	1.15 ± 0.01^c^
C17	n.d	n.d	n.d	n.d	n.d	n.d	1.26 ± 0.02^b^	1.95 ± 0.02^a^
C17:1	n.d	n.d	n.d	n.d	n.d	n.d	n.d	0.68 ± 0.01
C18	4.37 ± 0.01^f^	4.40 ± 0.05^f^	4.48 ± 0.02^f^	4.80 ± 0.02^e^	39.37 ± 0.08^a^	4.99 ± 0.06^d^	18.65 ± 0.09^c^	25.63 ± 0.04^b^
C18:1	38.47 ± 0.08^b^	37.80 ± 0.21^c^	35.95 ± 0.12^d^	31.70 ± 0.10^f^	0.15 ± 0.00^h^	39.92 ± 0.20^a^	28.80 ± 0.03^g^	32.35 ± 0.00^e^
C18:1 T	n.d	n.d	n.d	n.d	n.d	n.d	3.09 ± 0.02^b^	4.84 ± 0.04^a^
C18:2	10.60 ± 0.01^b^	8.68 ± 0.03^c^	8.40 ± 0.03^d^	8.06 ± 0.02^e^	0.05 ± 0.01^h^	20.79 ± 0.08^a^	1.78 ± 0.01^g^	2.11 ± 0.00^f^
C18:2 T	0.22 ± 0.06^d^	0.83 ± 0.01^a^	0.38 ± 0.03^c^	0.16 ± 0.00^d^	n.d	n.d	n.d	0.48 ± 0.00^b^
C18:3	0.29 ± 0.00^d^	0.18 ± 0.01^e^	0.24 ± 0.01^de^	0.23 ± 0.00^de^	n.d	1.82 ± 0.06^a^	0.55 ± 0.03^c^	1.06 ± 0.03^b^
C20	0.36 ± 0.01^cd^	0.40 ± 0.01^b^	0.38 ± 0.00^bc^	0.35 ± 0.01^d^	0.49 ± 0.02^a^	n.d	n.d	0.18 ± 0.01^e^
C20:1	0.02 ± 0.02^c^	0.03 ± 0.03^c^	0.03 ± 0.00^c^	0.01 ± 0.01^c^	n.d	n.d	0.82 ± 0.02^b^	0.91 ± 0.01^a^
C22	0.01 ± 0.01^a^	0.01 ± 0.02^a^	n.d	n.d	n.d	n.d	n.d	n.d
C24	0.01 ± 0.01^a^	n.d	n.d	n.d	n.d	n.d	n.d	n.d
SAFA (%)	52.27	50.17	59.69	54.75	99.65	31.04	60.56	55.87
USFA (%)	47.71	49.79	40.26	45.16	0.35	68.96	39.44	43.38
Iodine value	52.42 ± 0.71^a^	49.64 ± 0.30^b^	46.93 ± 0.13^c^	42.37 ± 0.29^d^	0.36 ± 0.02^h^	81.20 ± 0.14^a^	32.61 ± 0.05^g^	36.19 ± 0.04^f^

The values above are expressed in mean ± SD (n = 3). Different superscript letters (^a–h^) within the same row indicate significant differences (*p* < 0.05, Tukey's test). 33–36 = PS at MT of 33–36 °C; 38–42 = PS at MT of 38–42 °C; 44–46 = PS at MT of 44–46 °C; 45–49 = PS at MT of 45–49 °C; 55–60 = PS at MT of 55–60 °C; CF = Chicken fat; BF = Beef fat; MF = Mutton fat; n.d = not detected; SAFA = saturated fatty acid; USFA = unsaturated fatty acid.

The total unsaturation of the fat samples was further summarised by the iodine value (IV), which measures the degree of unsaturation and indicates the oxidative stability of the fat samples. Theoretically, fats with higher iodine values are less stable, softer in texture, more reactive, and more susceptible to oxidation and rancidification (Mata et al., 2014). CF, with the highest IV (81.20 ± 0.14), contained nearly 68.96 % unsaturated fatty acids in its fat composition. It was expected that 55–60 would have the lowest IV (0.36 ± 0.02) since its fatty acid profile has the most significant relative amounts of saturated fats (99.65 %). In the case of the remaining samples, the IV varies substantially; yet, the total unsaturated fatty acids fall within a relatively narrow range (36.35–48.57 %). Additionally, trace amounts of trans fatty acids (C18:1 t) were detected in beef fat (BF) and mutton fat (MF), which are naturally produced through anaerobic bacterial fermentation in the rumen of ruminant animals and are commonly present in animal and dairy products (Lichtenstein, 2003). This process involves the biohydrogenation of unsaturated fatty acids by rumen bacteria, resulting in various trans fatty acid isomers (Dewanckele et al., 2020). Unlike industrially produced trans fats, which have been linked to adverse health effects, naturally occurring trans fatty acids from ruminant sources are generally considered to have a different, less harmful health profile. In contrast, palm-based shortenings contained only negligible amounts of trans fat (C18:2 t), except for sample 55–60, which did not contain detectable trans fatty acids. Therefore, substituting animal fats with these palm-based shortenings is unlikely to raise significant concerns regarding trans fatty acid intake.

### 3.3 Solid fat content

**Fig. 1:**
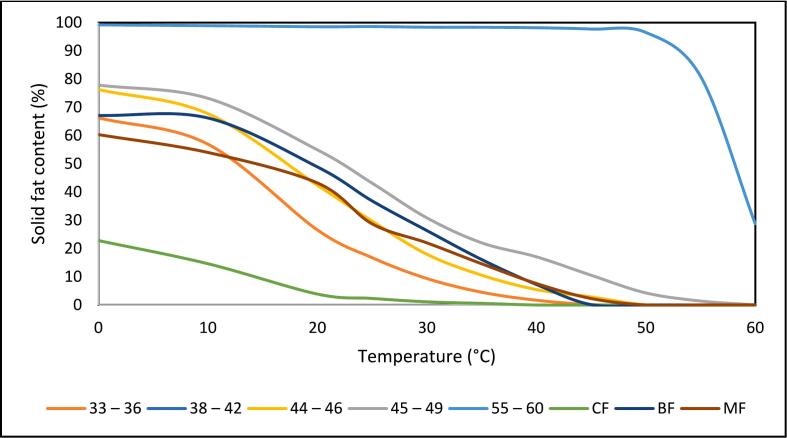
The solid fat content of palm-based shortenings (PS) of various melting temperatures (MT) and animal-based fats. 33–36 = PS at MT of 33–36 °C; 38–42 = PS at MT of 38–42 °C; 44–46 = PS at MT of 44–46 °C; 45–49 = PS at MT of 45–49 °C; 55–60 = PS at MT of 55–60 °C; CF = Chicken fat; BF = Beef fat; MF = Mutton fat.

The solid fat content (SFC) profiles reveal distinct patterns (Fig. 1) among the studied fats that warrant detailed comparative analysis. When examining the SFC profiles across different temperature ranges, three distinct clusters emerge: high-melting fats (55–60), intermediate-melting fats (38–42, 44–46, 45–49, BF, MF), and low-melting fats (CF). At lower temperatures (10 °C), sample 55–60 maintained exceptionally high solidity (98.87 %) compared to the intermediate group (ranging from 53.99 to 73.17 %), while CF exhibited the lowest SFC (14.6 %). At room temperature (25 °C), the differences became more pronounced, with sample 55–60 remaining predominantly solid (98.56 %), while the intermediate group showed moderate solidity (16.75–43.14 %), appropriate for spreadable products. Most notably, at body temperature (35 °C), only sample 55–60 maintained substantial solidity (98.31 %), while the intermediate group showed values ranging from 4.52 to 22.02 %, and CF was almost completely melted (0.58 %).

From an applications perspective, the comparative analysis highlights distinct fat samples suited for different food systems based on their SFC profiles. Sample 55–60 exhibits exceptional thermal stability, maintaining very high SFC values near 98 % at 25 °C and 35 °C. This makes it ideal for bakery products that require structural integrity at elevated temperatures and in warm storage conditions. In contrast, samples 38–42, 44–46, and 45–49 show gradual decreases in SFC from refrigeration (10 °C) to room temperature (25 °C), displaying melting behaviour similar to beef and mutton fat. This suggests that these palm-based shortenings could effectively serve as alternatives in food applications, such as certain traditional meat-based products, that require fats with good plasticity at low temperatures and sufficient firmness at room temperature.

In addition, the data indicate a strong relationship between the amount of SAFAs and the SFC profiles across all samples, regardless of their source. This observation aligns with and extends the findings of Sahri and Dian (2011), who reported such a relationship for fats derived from the same source. In particular, samples with higher SAFA content consistently exhibit higher SFC values at 25 °C, suggesting that SAFA content is a key factor influencing fat functionality at room temperature. This relationship is further supported by the fatty acid profiles in Table 2 and the corresponding SFC profiles shown in Fig. 1 and Table 3. These findings demonstrate that SFC profiles and fatty acid composition are crucial when selecting suitable fats for specific food applications.

**Table 3:** Solid fat contents of palm-based shortenings (PS) of various melting temperatures (MT) and animal-based fats.

Temperature (°C)	33–36	38–42	44–46	45–49	55–60	CF	BF	MF
0	66.26 ± 0.29^e^	69.67 ± 0.64^d^	76.26 ± 0.09^c^	77.85 ± 0.11^b^	99.15 ± 0.23^a^	22.81 ± 1.08^g^	67.10 ± 0.20^e^	60.32 ± 0.09^f^
10	56.93 ± 0.39^f^	61.13 ± 0.16^e^	67.73 ± 0.32^c^	73.17 ± 0.139^b^	98.87 ± 0.06^a^	14.60 ± 0.23^h^	66.20 ± 0.06^d^	53.99 ± 0.14^g^
20	26.57 ± 0.42^e^	32.02 ± 5.89^e^	42.40 ± 0.20^d^	54.92 ± 0.27^b^	98.50 ± 0.20^a^	3.87 ± 0.03^f^	48.88 ± 0.05^c^	43.20 ± 0.09^cd^
25	16.75 ± 0.22^d^	24.33 ± 0.04^c^	29.67 ± 0.24^c^	43.14 ± 0.28^b^	98.56 ± 0.02^a^	2.32 ± 0.07^e^	36.85 ± 0.05^b^	28.73 ± 6.65^c^
30	9.37 ± 0.356^g^	14.89 ± 0.20^f^	17.97 ± 0.24^e^	30.85 ± 0.25^b^	98.33 ± 0.03^a^	1.12 ± 0.17^h^	26.35 ± 0.13^c^	22.00 ± 2.81^d^
35	4.52 ± 0.26^g^	8.36 ± 0.19^f^	10.56 ± 0.18^e^	22.02 ± 0.26^b^	98.31 ± 0.02^a^	0.58 ± 0.04^h^	16.11 ± 0.14^c^	14.60 ± 0.17^c^
40	1.69 ± 0.16^e^	5.13 ± 0.20^d^	5.46 ± 0.11^d^	17.06 ± 0.45^b^	98.14 ± 0.57^a^	n.d	7.27 ± 0.06^c^	7.61 ± 0.11^c^
45	0.14 ± 0.06^e^	1.97 ± 0.11^d^	2.85 ± 0.20^c^	10.54 ± 0.04^b^	97.68 ± 0.59^a^	n.d	0.13 ± 0.03^e^	2.30 ± 0.17^cd^
50	0.001 ± 0.002^c^	0.45 ± 0.62^c^	0.04 ± 0.05^c^	4.28 ± 0.32^b^	96.49 ± 0.08^a^	n.d	0.02 ± 0.04^c^	n.d
55	0.00 ± 0.00^b^	0.01 ± 0.01^c^	n.d	1.41 ± 0.10^b^	80.98 ± 0.46^a^	n.d	n.d	n.d
60	n.d	0.00 ± 0.00^b^	n.d	0.03 ± 0.01^b^	28.76 ± 0.56^a^	n.d	n.d	n.d

The values above are expressed in mean ± SD (n = 3). Different superscript letters (^a–h^) within the same row indicate significant differences (*p* < 0.05, Tukey's test). 33–36 = PS at MT of 33–36 °C; 38–42 = PS at MT of 38–42 °C; 44–46 = PS at MT of 44–46 °C; 45–49 = PS at MT of 45–49 °C; 55–60 = PS at MT of 55–60 °C; CF = Chicken fat; BF = Beef fat; MF = Mutton fat.

### 3.4 Colour profile and visual appearance

The colour profile of the palm-based shortening and animal fat samples is expressed using the Lovibond colour parameters, specifically red, yellow, and white. Lovibond colour assessment is a crucial step in the refining process of the oils and fats industry, as it indicates when the target colour has been achieved and when the refining process can be halted. As shown in Table 4, the redness and whiteness indices of the samples did not vary significantly, falling within ranges of 0.9–2.0 and 0.1–1.0, respectively. The whiteness of samples 33–36, 55–60, and BF cannot even be detected. In contrast, yellowness denotes a relatively wide range (2.0–40.0), with some samples being more intense than others. The visual appearance of all fat samples, both in solid and melted forms, can be observed in Fig. 2. The appearances of the palm-based shortening samples were comparable to those of shortenings derived from palm-based fats that are available in the market and reported in the literature (Perez-Santana et al., 2022). The melted version exhibited more intense yellowness compared to the solid fat samples. This can be explained by melting the solid fats, which makes them more transparent, allowing more light to pass through and intensifying the colour. The colours of the solid forms of MF, CF, and 33–36 visibly appear more intense, while the rest appear lighter, with a creamy appearance slightly tainted with yellow. This observation also aligns with the visual appearance of the liquid form of the fat samples. Visually, the palm-based shortenings and the animal fats exhibit striking similarities in their liquid and solid states, rendering them suitable substitutes for animal fats in food production applications. (See Fig. 2.)

**Table 4:** Lovibond colour profiles and thermal properties of palm-based shortenings (PS) of various melting temperatures (MT) and animal-based fats.

	Lovibond colour profiles			SMP (°C)	DSC thermograms peaks			
					1nd melting peak		2nd melting peak	
	Red	Yellow	White		T_p_ (°C)	∆H (J/g)	T_p_ (°C)	∆H (J/g)
**33–36**	2.0	40.0	nd	38.43 ± 0.06^g^	5.74	39.95	37.71	20.84
**38–42**	2.0	19.0	1.0	41.43 ± 0.06^e^	6.51	56.20	47.41	15.68
**44–46**	2.0	21.0	0.1	43.17 ± 0.06^d^	7.10	41.15	45.73	19.33
**45–49**	2.1	19.0	0.1	49.93 ± 0.06^b^	5.16	28.78	51.50	28.62
**55–60**	2.0	20.0	nd	58.43 ± 0.12^a^	n.d	n.d	62.91	203.56
**CF**	0.9	2.0	0.5	29.00 ± 0.10^h^	3.80	49.07	31.45	26.57
**BF**	2.1	20.1	nd	40.77 ± 0.25^f^	6.40	15.01	48.28	38.36
**MF**	2.0	9.6	0.1	44.63 ± 0.12^c^	2.84	35.87	41.14	20.64

Only values for SMP are expressed in mean ± SD (n = 3). Different superscript letters (^a–h^) within the same column indicate significant differences (*p* < 0.05, Tukey's test). SMP = solid melting point; DSC = differential scanning calorimetry; Tp = peak temperature; n.d = not detected; 33–36 = PS at MT of 33–36 °C; 38–42 = PS at MT of 38–42 °C; 44–46 = PS at MT of 44–46 °C; 45–49 = PS at MT of 45–49 °C; 55–60 = PS at MT of 55–60 °C; CF = Chicken fat; BF = Beef fat; MF = Mutton fat.

**Fig. 2:**
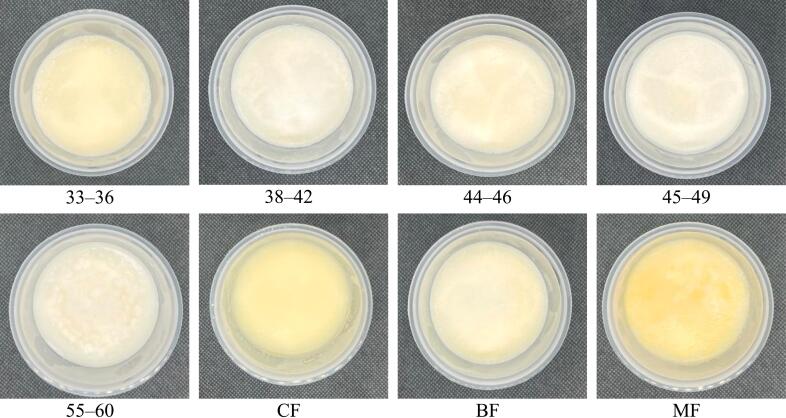
**(a).** The images of solid palm-based shortenings (PS) of various melting temperatures (MT) and animal-based fats. 33–36 = PS at MT of 33–36 °C; 38–42 = PS at MT of 38–42 °C; 44–46 = PS at MT of 44–46 °C; 45–49 = PS at MT of 45–49 °C; 55–60 = PS at MT of 55–60 °C; CF = Chicken fat; BF = Beef fat; MF = Mutton fat.

**Fig. 2:**
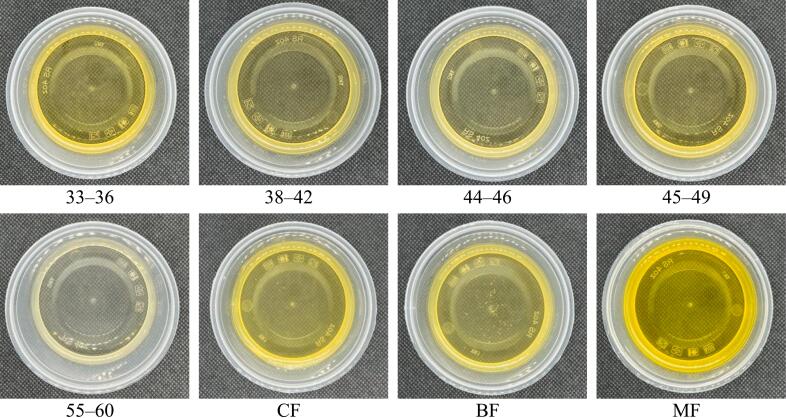
**(b).** The images of liquid palm-based shortenings (PS) of various melting temperatures (MT) and animal-based fats. 33–36 = PS at MT of 33–36 °C; 38–42 = PS at MT of 38–42 °C; 44–46 = PS at MT of 44–46 °C; 45–49 = PS at MT of 45–49 °C; 55–60 = PS at MT of 55–60 °C; CF = Chicken fat; BF = Beef fat; MF = Mutton fat.

### 3.5 Thermal characteristics (Slip Melting Point and Differential Scanning Calorimetry)

Slip melting point (SMP) and differential scanning calorimetry (DSC) are two different techniques used to study the thermal behaviour of fat samples. SMP is a more straightforward method for determining the temperature at which a column of fat samples in an open capillary tube begins to rise upon heating under specific conditions (Association of Official Analytical Chemists (AOAC), 2017). In contrast, DSC is a more complex thermal analysis technique that measures the heat flow of a sample as a function of temperature, providing more comprehensive information to explain the thermal behaviour of fat samples.

Table 4 displays the solidifying melting point (SMP) of the palm-based shortening and animal fat samples. The SMP of all fat samples (29.00–58.43) differed significantly based on their specific melting temperatures. Higher SMPs were recorded in samples with a greater concentration of saturated fatty acids. Sample 55–60, which contains a high composition of saturated fatty acids, had a solidifying melting point of 58.43 °C, resulting in a hard, solid form at room temperature. This feature is advantageous for creating stable emulsions in emulsified meat products, essential for maintaining texture and preventing fat separation during cooking (Faridah et al., 2023). In contrast, CF, high in unsaturated fatty acids, exhibited a low SMP of 29.

**Fig. 3:**
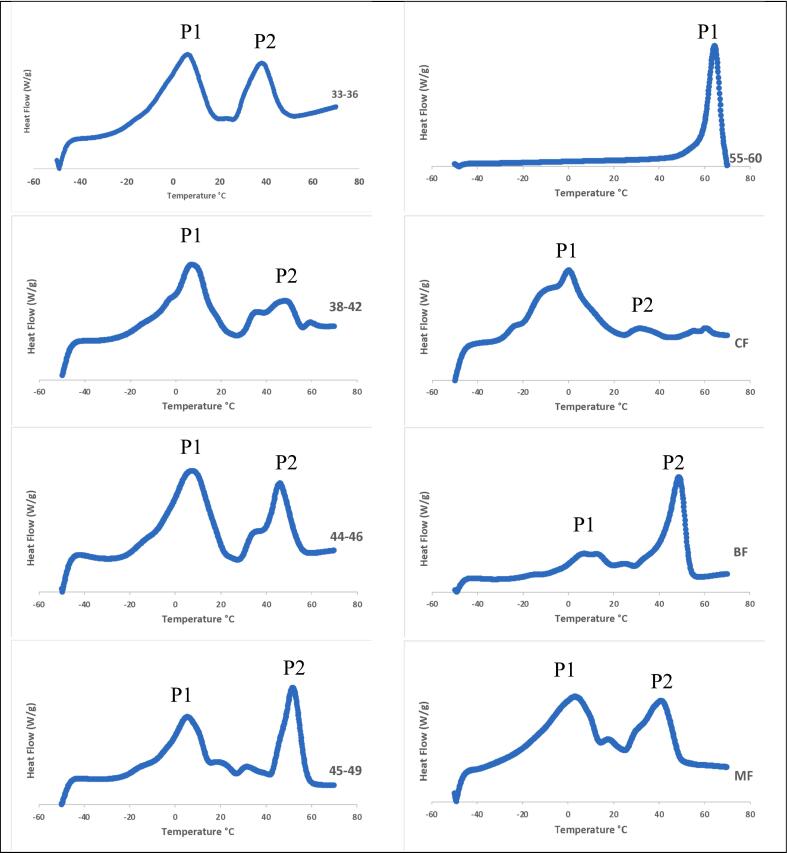
The DSC thermograms of palm-based shortenings (PS) of various melting temperatures (MT) and animal-based fats. 33–36 = PS at MT of 33–36 °C; 38–42 = PS at MT of 38–42 °C; 44–46 = PS at MT of 44–46 °C; 45–49 = PS at MT of 45–49 °C; 55–60 = PS at MT of 55–60 °C; CF = Chicken fat; BF = Beef fat; MF = Mutton fat.

The DSC curves in Fig. 3 show typical heating thermograms of solid fat samples that provide vital information, specifically the onset, peak, and end temperatures, peak height, and enthalpy, which are very useful for understanding the thermal behaviours of the samples. The DSC curve of the samples during heating exhibits endothermic peaks that correspond to the melting of the fat samples. The palm shortenings and animal fat samples, except for sample 55–60, exhibit two well-distinguishable peaks: the low-temperature endotherm (olein fraction) and the high-temperature endotherm (stearin fraction). These observations align with the thermographs of different types of palm oil and palm stearin-based shortenings reported by Abdul Azis et al. (2011). The single peak shown by sample 55–60 can be explained by the fact that this sample has a very low amount of olein fractions (oleic and linoleic acids), which is supported by the data on fatty acid composition (Table 2). The presence of one or multiple peaks on a melting curve can be attributed to various factors, including impurities, sample composition, or polymorphisms and isomers with different melting points (Kumar & Aravind, 2021).

The peak heights and melting enthalpy values of the high-temperature endothermic melting events of the palm shortenings and animal fat samples were measured, and their values are presented in Table 4. The peak height indicates the sample's melting point, while the enthalpy value measures the amount of heat absorbed by the sample during a thermal event. As demonstrated by the graph, the peak height and enthalpy value of sample 55–60 exhibited significantly higher trends than those of the other palm shortenings and animal fat samples. These observations indicate that this sample displayed superior structural stability with 87 %, 81 %, and 89 % higher melting enthalpy (ΔH = 203.56 J/g) and temperature elevations of 31.4 °C, 48.3 °C, and 41.1 °C in peak melting temperature compared to CF, BF, and MF, respectively. These enhanced thermal properties correlate with its increased SFC (Table 3), which improves heat resistance during food processing and storage, facilitates better fat network formation for texture maintenance, and delays oil separation in final food products.

### 3.6 Crystalline morphology (X-ray Diffraction)

Polymorphism is regarded as the vital criterion for evaluating the physical characteristics of fats relating to sensory quality and lubrication. There are mainly three types of polymorphic forms of fats: α (alpha), β’ (beta prime), and β (beta). These polymorphic crystal forms can be identified using short spacing in X-ray diffraction patterns. The characteristics of the α and β forms are demonstrated at short spacing lines of 4.15 Å and 4.6 Å, respectively, while the β’ form appears at three short spacing lines of 3.8 Å, 4.2 Å, and 4.3 Å (Fu et al., 2018).

The diffractogram in Fig. 4(a) presents the polymorphic forms of all palm-based shortening and animal fat samples. Samples 33–36, 38–42, 44–46, 45–49, and CF show a similar pattern, with a major diffraction peak at 4.6 Å, indicative of the β polymorph. Several noticeable peaks at a short spacing of 3.8–4.4 Å were also detected, corresponding to the β’ polymorph. Meanwhile, samples 55–60, BF, and MF displayed unique patterns with distinctive diffraction peaks. The 55–60 sample exhibited a relatively major peak at 3.85 Å, while some other peaks were small and negligible. This short spacing pattern indicates that β is the dominant form in the 55–60 sample. The BF sample shows the three strongest peaks at 3.85, 4.2, and 4.6 Å, while the MF sample shows the three strongest peaks at 4.4, 4.6, and 4.8 Å. The peak intensities are very similar, making it difficult to conclude the dominant polymorph in this sample. Consequently, the area under the corresponding short spacing peaks was measured to determine the relative content (%) of the polymorphic forms, which allows for the estimation of the level of each polymorph form in all samples. The remaining minor peaks were disregarded. Fig. 4(b) presents the findings. (See Fig. 4.)

**Fig. 4:**
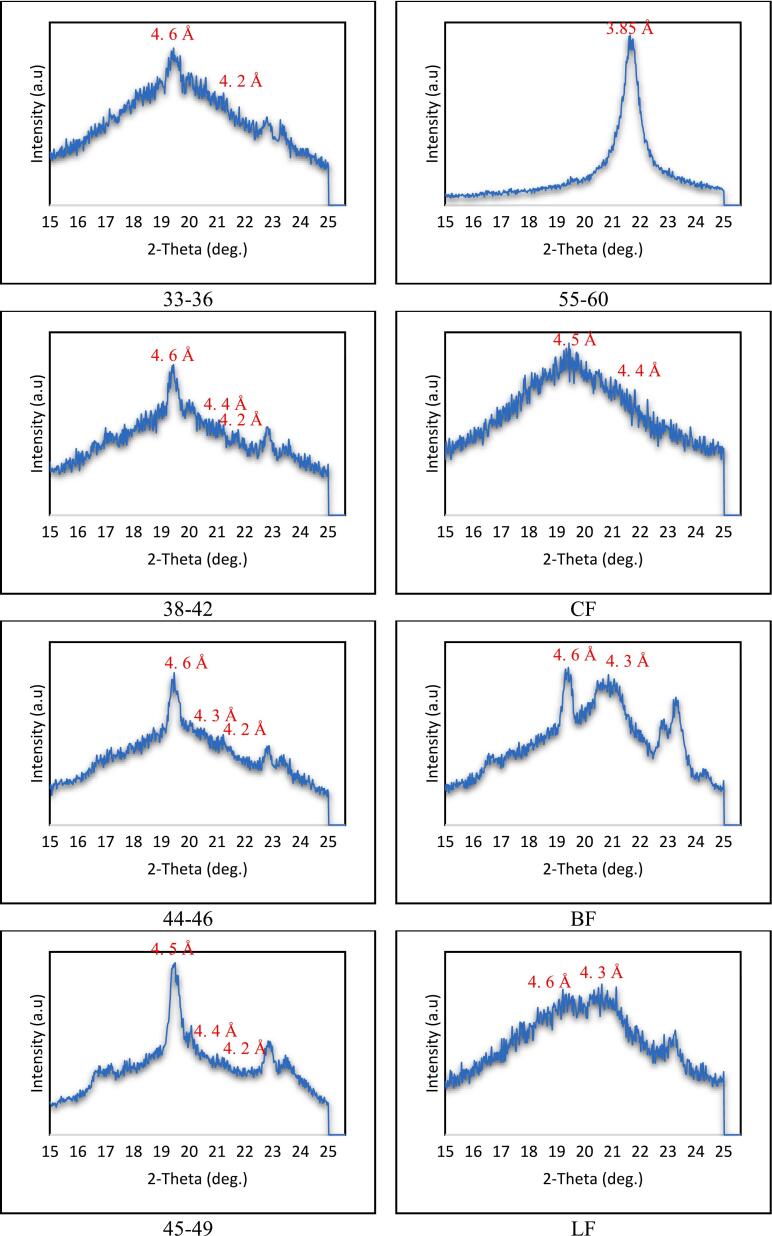
**(a).** The XRD patterns of palm-based shortenings (PS) of various melting temperatures (MT) and animal-based fats. 33–36 = PS at MT of 33–36 °C; 38–42 = PS at MT of 38–42 °C; 44–46 = PS at MT of 44–46 °C; 45–49 = PS at MT of 45–49 °C; 55–60 = PS at MT of 55–60 °C; CF = Chicken fat; BF = Beef fat; MF = Mutton fat.

**Fig. 4:**
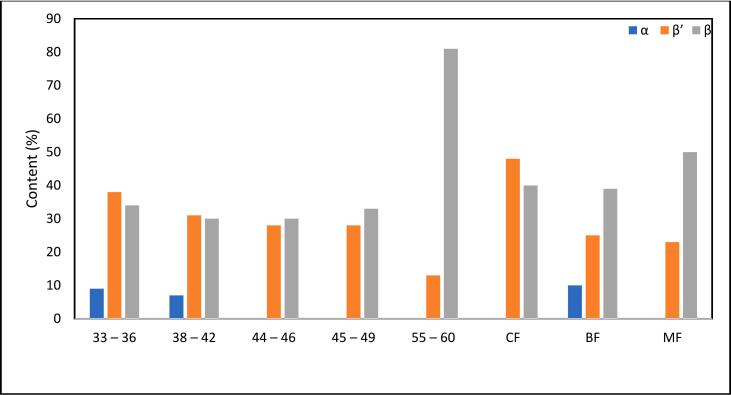
**(b).** The relative content (%) of α, β, and β″ polymorphism in the palm-based shortenings (PS) of various melting temperatures (MT) and animal-based fats. 33–36 = PS at MT of 33–36 °C; 38–42 = PS at MT of 38–42 °C; 44–46 = PS at MT of 44–46 °C; 45–49 = PS at MT of 45–49 °C; 55–60 = PS at MT of 55–60 °C; CF = Chicken fat; BF = Beef fat; MF = Mutton fat.

As for the shortening samples, the relative content of the β form increased as the melting temperatures of the samples increased. This is due to the higher molecular packing density of the β form compared to other crystal polymorphs (Ribeiro et al., 2015). This denser packing arrangement in the β form leads to stronger intermolecular bonds and interactions between fat molecules, resulting in increased stability and a higher melting point (Hondoh & Ueno, 2016). While the β form is the most stable, it is undesirable at higher contents due to the large crystal size, which results in a rough and gritty texture. On the other hand, β’ crystals are relatively small and have smoother textures, providing good aeration, softer textures, and creaminess properties (Campioni et al., 2021), making them excellent for products such as shortenings. CF was dominated by β’ crystal for the animal fat samples, whereas β crystal dominated BF and MF. Palm shortenings with lower melting points (e.g., samples 33–36, 38–42) predominantly form β’ crystals, which are small and smooth-textured, mirroring the desirable properties of animal fats like CF. The polymorphic behaviour of β’ ensures comparable aeration, creaminess, and mouthfeel in baked or fried foods (Yılmaz & Öğütcü, 2015). This structural compatibility enables palm-based shortenings to serve as viable alternatives to animal fats, maintaining the sensory and functional quality of end products.

### 3.7 Similarity-based classification by PCA

PCA is a statistical method used to summarise the information content in extensive data by reducing noise and complexity while highlighting the most important features and relationships among the studied variables. In the present study, PCA was employed to visualise the similarities between palm shortening and animal fat samples by summarising the triplicate data from physicochemical analyses. Before running the PCA, normalising the data is essential to avoid bias toward variables with larger scales, ensuring that all features contribute equally to the analysis. Data normalisation also enables PCA to accurately identify the principal components that best represent the variance in the dataset without skewing results and leading to inaccurate interpretations of the data's variance (Dutt, 2021). Moreover, a Kaiser-Meyer-Olkin (KMO) test, which measures data sampling adequacy, must also be conducted before the PCA. An adequate dataset is crucial to determine whether the generated model can extract dormant variables from the examined dataset. Other researchers have established that the minimum KMO test value should be 0.5, while the ideal is above 0.8 (Kaiser, 1974; Williams et al., 2010). The KMO test value for this study is 0.568, which barely meets the minimum requirement, but the data is sufficient to perform the PCA.

**Fig. 5:**
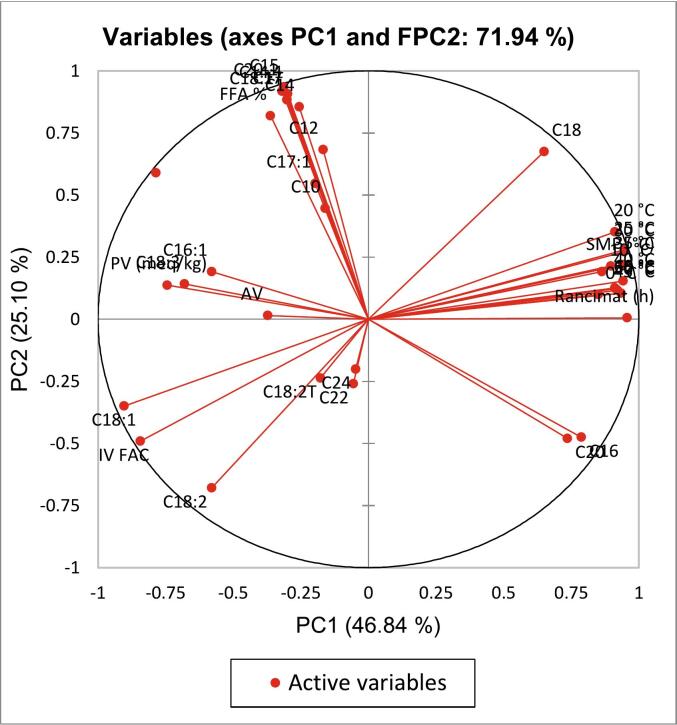
The variables loading plot of principal component (PC) analysis showing contributions of the physicochemical properties of palm-based shortenings of various melting temperatures and animal-based fats.

**Fig. 6:**
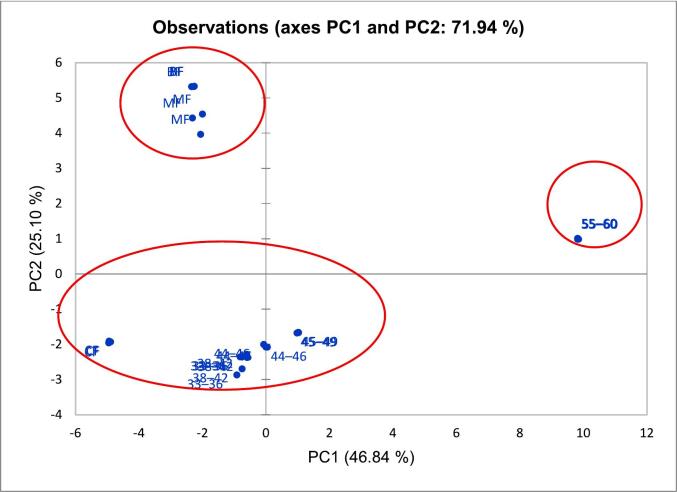
The score plot of principal component (PC) analysis model using physicochemical properties data of palm-based shortenings of various melting temperatures and animal-based fats. 33–36 = PS at MT of 33–36 °C; 38–42 = PS at MT of 38–42 °C; 44–46 = PS at MT of 44–46 °C; 45–49 = PS at MT of 45–49 °C; 55–60 = PS at MT of 55–60 °C; CF = Chicken fat; BF = Beef fat; MF = Mutton fat.

Fig. 5 and Fig. 6 illustrate the loading plot and score plot, respectively, for the palm-based shortening and animal fat samples, depicting the projection of their physicochemical properties data onto two Principal Components (PCS) dimensions. The analysis of the dataset in this study using PCA revealed 5 PCs with eigenvalue (EV) > 1, which accounted for 94.51 % cumulative variability (CV) of the dataset. However, only the score plot of the PCs with the highest CVs (PC1 and PC2) was interpreted because these two PCs captured the most significant amount of variance and are sufficient for uncovering meaningful insights into the dataset. Accordingly, PC1 and PC2 explained 71.94 % of CV at an EV of 9.29 in this study (Table 5).

**Table 5:** Principal components (PCs) information interpreted from Principal component analysis of the physicochemical properties data of palm-based shortenings of various melting temperatures and animal-based fats.

	PC1	PC2	PC3	PC4	PC5
Eigenvalue	17.33	9.29	4.28	2.75	1.32
Variability (%)	46.84	25.10	11.58	7.42	3.57
Cumulative variability (%)	46.84	71.94	83.52	90.94	94.51

The variables loading plot (Fig. 5), which visualises the contributions of the original physicochemical variables to the principal components, reveals important insights into the relationships among variables and their influence on sample differentiation. The angles between vectors in the loading plot indicate variable correlations: small angles signify a strong positive correlation, angles near 90° indicate little to no correlation, and large angles close to 180° represent a strong negative correlation. In the loading plot's correlation circle, variables such as solid fat contents at 20 °C, 30 °C, and 40 °C, together with Rancimat values (which indicate oxidative stability), cluster on the positive side of F1. This clustering suggests these variables are strongly correlated and significantly distinguish palm-based shortenings with higher melting points. Conversely, peroxide value and anisidine value, both indicators of fat oxidation and quality deterioration, group on the negative side of F1, indicating an inverse relationship with solid fat content and oxidative stability. The fatty acid composition variables are dispersed around the circle, with saturated fatty acids (e.g., C18) positioned opposite to unsaturated fatty acids (e.g., C18:2), reflecting their well-known negative correlation. The iodine value, a measure of unsaturation, aligns closely with the unsaturated fatty acids on the negative side of F1, reinforcing the interpretation of these relationships. Overall, the loading plot effectively captures the complex interrelationships among physicochemical properties and highlights how solid fat content, fatty acid composition, and oxidative stability are key factors driving sample differentiation.

Building on this, the score plot (Fig. 6) reveals clear groupings of data points, indicating the presence of distinct physicochemical characteristics exhibited by the samples within the dataset. The score plots show that all palm-based shortening and animal fat samples can be classified into three different groups: (1) BF and MF, (2) 33–36, 38–42, 44–46, 45–49, and CF, and (3) 55–60 only. Notably, chicken fat (CF) clusters closely with most palm-based shortening samples (33–36, 38–42, 44–46, 45–49), suggesting similarities in their physicochemical profiles. Additionally, the close distance shared by the samples in the score plot indicates that they share some similarities with the studied matrix variables (Rohman et al., 2012). Thus, this observation underscores the potential for these palm-based shortenings to mimic the functional properties of CF, thereby supporting their use as viable substitutes in sustainable food production. Conversely, the sample 55–60 is an outlier due to its significantly different physicochemical profile, which diverges from the general trends observed among the other samples.

The combined interpretation of the loading and score plots underscores the importance of specific physicochemical parameters in differentiating the samples. The 55–60 °C shortening separation along PC1 can be attributed to its elevated melting point and lower unsaturation, as reflected in the loading plot variables. Meanwhile, the clustering of CF with other palm-based shortening samples (33–36, 38–42, 44–46, 45–49) suggests that these plant-based fats can mimic the functional properties of chicken fat, supporting their use as sustainable alternatives in food production. Conversely, the distinct profile of the 55–60 °C sample highlights the need to select palm-based shortenings carefully based on intended application requirements. In summary, these findings emphasise how PCA effectively reveals key physicochemical traits that guide the selection of palm-based shortenings as viable and tailored alternatives to animal fats in various food applications.

## 4 Conclusion

In conclusion, this study presents a comprehensive comparative analysis of the physicochemical properties of various melting temperatures of palm-based shortenings with animal fats. The high-melting palm-based shortening (55–60 °C) offers superior oxidative stability and thermal resilience, making it ideal for high-temperature processing. Meanwhile, the palm-based shortenings with lower melting temperatures (33–49 °C) mirror the crystal structures of animal fats, providing comparable textural properties. Colour and visual assessments confirm that these palm-based shortenings are visually similar to animal fats in both solid and melted forms, enhancing their acceptability as direct substitutes. Principal component analysis further supports these findings by illustrating clear groupings based on key physicochemical parameters, with several palm-based shortenings clustering closely with chicken fat. Thus, palm-based shortenings with the melting temperatures of 33–49 °C offer a promising, sustainable alternative to animal fat, specifically chicken fat, in food applications.

## CRediT authorship contribution statement

**Mohd Razali Faridah:** Writing – original draft, Visualization, Software, Resources, Project administration, Methodology, Investigation, Formal analysis, Data curation, Conceptualization. **Amelia Najwa Ahmad Hairi:** Software, Methodology, Investigation, Formal analysis, Data curation. **Masni Mat Yusoff:** Validation, Supervision. **Ashari Rozzamri:** Validation, Supervision. **Wan Zunairah Wan Ibadullah:** Validation, Supervision. **Mohammad Rashedi Ismail-Fitry:** Writing – review & editing, Validation, Supervision, Funding acquisition, Conceptualization.

## Declaration of generative AI and AI-assisted technologies in the writing process

While preparing this work, the authors utilised Perplexity AI to aid in writing by generating text suggestions, enhancing clarity, and improving the overall quality of the written content. After employing this tool/service, the authors reviewed and edited the content as necessary and take full responsibility for the content of the published article.

## Declaration of competing interest

The authors declare the following financial interests/personal relationships which may be considered as potential competing interests: Author Amelia Najwa Ahmad Hairi was employed by the company SD Guthrie Technology Centre Sdn. Bhd. The remaining authors declare that no conflicts of interest, whether commercial or financial, influenced the research.

## Data Availability

Data will be made available on request.
